# Metabolite profiling of *Bacillus velezensis* DM extract linked to the rhizosphere of *Datura metel* L. and dermatoprotective potential of an isolated glycoglycerolipid

**DOI:** 10.1186/s12934-026-02927-w

**Published:** 2026-02-02

**Authors:** Mohamed A. Awad, Sherif F. Hammad, Fahd M. Abdelkarem, Amira Elkattan, Samir F. El-Mashtoly, Hesham S. M. Soliman, Kuniyoshi Shimizu

**Affiliations:** 1 Botany and Microbiology Department, Faculty of Science, Sohag University, Sohag, 82524 Egypt; 2 Department of Pharmaceutical Chemistry, Faculty of Pharmacy, Helwan University, Cairo, 11795 Ain-Helwan Egypt; 3 Department of Pharmacognosy, Faculty of Pharmacy, Al-Azhar University, Assiut, 71524 Egypt; 4 Department of Agro-Environmental Sciences, Graduate School of Bioresource and Bioenvironmental Sciences, Kyushu University, Fukuoka, 819-0395 Japan; 5 Department of Pharmacognosy, Faculty of Pharmacy, Mansoura University, Mansoura, 35516 Egypt; 6 Leibniz Institute of Photonic Technology, Albert-Einstein-Straße, 07745 Jena, Germany; 7 Department of Pharmacognosy, Faculty of Pharmacy, Helwan University, Ain-Helwan, Cairo, 11795 Egypt; 8 Faculty of Pharmacy, Egypt-Japan University of Science and Technology (E-JUST), New Borg El-Arab City, 21934 Alexandria Egypt; 9 Kyushu University Institute for Asian and Oceanian Studies, Fukuoka, 819-0395 Japan

**Keywords:** *Datura metel* L., Rhizosphere, *Bacillus velezensis* DM, LC-MS, Microbial glycolipids, Cosmeceutical activities

## Abstract

**Background:**

Rhizosphere bacterial metabolites play a pivotal role in drug discovery by producing diverse bioactive compounds with cosmeceuticals and pharmaceuticals applications, offering eco-friendly and potent alternatives for skin care, wound healing, and therapeutic formulations. This study outlines the procedures for fermenting and processing *Bacillus velezensis* DM, derived from the rhizosphere of *Datura metel* L., to isolate secondary metabolites. Chemical profiling was performed to analyze and identify the bacterial metabolites using HR-LC-MS. The EtOAc extract obtained from the cultured strain underwent fractionation and purification using a range of chromatographic techniques. The isolated compound was then structurally characterized through 1D, 2D-NMR and HR/MS. Bacterial extract and its pure compound were assessed for their anti-phototoxicity using MTT assay. Furthermore, the anti-melanogenesis and anti-allergic activities of the isolated compound were evaluated using melanin content inhibition assays in B16-F10 melanoma cells and β-hexosaminidase release assays in RBL-2H3 cells, respectively.

**Results:**

Chemical profiling of the crude extract of *Bacillus velezensis* DM indicated the presence of 46 compounds. The main metabolites present in bacterial extract were nitrogenous compounds, dipeptides and lipid derivatives. A microbial glycoglycerolipid derivative, (2 S)-1-O-(9Z,12Z-octadecadienoyl)-3-O-*β*-D-galactopyranosylglycerol, was successfully isolated from the strain. The crude bacterial extract exhibited high cytotoxicity toward NHDF cells, reducing viability to 56.3% even at the lowest tested concentration. It also showed no photoprotective activity, as cell viability following UVB exposure remained below that of the UVB-only control (39.9%). In contrast, the purified glycoglycerolipid compound was non-cytotoxic and even appeared to promote HaCaT keratinocyte viability. However, it did not confer protection against UVB-induced damage. The compound exhibited a dose-dependent reduction in melanin levels, with a maximum inhibition of 27% observed at 5 µM. However, the melanin suppression trend closely mirrored a decrease in cell viability. Although the glycoglycerolipid compound improved RBL-2H3 cell viability, it did not significantly inhibit β-hexosaminidase release, suggesting no effective anti-allergic activity.

**Conclusion:**

These findings demonstrate the potential of *Bacillus velezensis* DM as a microbial source of bioactive glycoglycerolipids. Further structural optimization and mechanistic investigation are required to enhance its functional specificity for safe and effective dermatological or cosmeceutical applications.

**Supplementary Information:**

The online version contains supplementary material available at 10.1186/s12934-026-02927-w.

## Background

Bacterial natural products have garnered increasing attention in recent years for their multifaceted bioactivities relevant to cosmeceutical and pharmaceutical applications. These microbial metabolites exhibit significant potential in drug discovery due to their antimicrobial, anti-inflammatory, and enzyme-inhibitory properties. Specifically, compounds isolated from *Bacillus subtilis* and related strains have shown remarkable photoprotective effects, making them promising candidates for inclusion in sunscreens and anti-aging skincare formulations [1, 2]. Moreover, these natural products display notable anti-melanogenesis activity through tyrosinase inhibition, contributing to skin-whitening and anti-hyperpigmentation treatments [3]. Additionally, their anti-allergic properties, linked to mast cell stabilization and histamine suppression, highlight their therapeutic relevance in allergic dermatitis and related conditions [4]. This broad bioactivity spectrum underscores the role of *Bacillus*-derived substances as vital tools in developing innovative and natural-based dermatological and therapeutic solutions.

*Datura metel* L., a well-known member of the Solanaceae family, is pharmacologically significant due to its rich profile of tropane alkaloids such as atropine, scopolamine, and hyoscyamine. Recent studies have highlighted its antioxidant and anti-inflammatory activities through inhibition of nitric oxide and prostaglandin pathways, making it promising for managing inflammatory and neurodegenerative disorders [5, 6]. Additionally, *D. metel* L. extracts have shown potential in wound healing and antimicrobial activity, attributed to synergistic actions of its alkaloids and phenolics [7, 8]. While these properties underline its traditional ethnomedicinal value, standardized extraction and toxicity evaluation remain crucial for therapeutic applications.

Among *Bacillus* species, *B. velezensis* is widely recognized for its ability to synthesize bioactive metabolites with antimicrobial and biocontrol functions. However, its metabolic potential remains largely uncharacterized in unique rhizospheric niches. The rhizosphere of *Datura metel* L., a plant rich in alkaloids and phenolics, provides a distinctive microenvironment that may influence bacterial secondary metabolism. Such interaction-driven modulation could result in novel metabolites with dermatoprotective or cosmeceutical potential. Therefore, this study specifically investigates *B. velezensis DM*, isolated from the *D. metel* L. rhizosphere, to explore its metabolite profile and the dermatoprotective relevance of its isolated glycoglycerolipid compound [9].

*Bacillus velezensis* strains isolated from the rhizosphere are gaining significant attention as powerful plant growth-promoting rhizobacteria (PGPR). Recent studies have unveiled their ability to produce a diverse array of secondary metabolites, such as surfactin, iturin, fengycin, and difficidin, which inhibit a broad range of plant pathogens while simultaneously enhancing plant health and stress tolerance [6, 10]. Genomic investigations have confirmed the presence of gene clusters responsible for these metabolites, providing molecular insights into their antagonistic and symbiotic behaviors [9, 11]. The multi-trait performance of these strains in biocontrol, growth enhancement, and environmental resilience positions *B. velezensis* as a key agent in sustainable agriculture. The present study builds upon prior work in which the authors isolated *Bacillus velezensis* strain DM from the rhizosphere of *Datura metel* L. and performed molecular identification through 16 S rRNA gene sequencing [6].

Glycolipids are natural biosurfactants produced by various bacterial species, such as *Pseudomonas*, *Bacillus*, and *Rhodococcus*, known for their amphiphilic structure and eco-friendly profile. As secondary metabolites, they exhibit diverse bioactivities, including antimicrobial, anti-inflammatory, wound-healing, and anticancer, making them attractive candidates in both pharmaceutical and cosmeceutical applications [12]. In wound healing, rhamnolipid-based nanoformulations have demonstrated enhanced cell migration and stability in dermal applications, underlining their biomedical utility [13].

The current study aimed to isolate and characterize a glycoglycerolipid compound from the EtOAc fraction of *Bacillus velezensis* DM, obtained from the rhizosphere of *Datura metel* L. Additionally, chemical profiling was conducted to identify various components within the bacterial extract. The skin-protective effect of this extract was evaluated in comparison to the purified compound under UV radiation exposure. Lastly, the study investigated the potential bioactivities of the isolated compound, including its cytotoxic, anti-allergic and anti-melanogenesis properties. Based on the unique chemical and ecological context of the rhizosphere-associated *Bacillus velezensis* DM, we hypothesize that this strain produces structurally diverse secondary metabolites, including glycolipid derivatives, that contribute to distinct dermatoprotective bioactivities such as anti-melanogenic and cytoprotective effects.

## Materials and methods

### Materials

Analytical-grade organic solvents, including MeOH, ACN, DCM, EtOAc, DMSO, and FA, were procured from Wako Pure Chemical Industries (Osaka, Japan). The chromatographic resin employed in this study was silica gel 60 (Merck, Germany) and thin-layer chromatography (TLC) employing DC-Alufolien plates coated with silica gel 60 F254 (Merck, Germany).

### Collection of bacterial strain

The bacterial strain *Bacillus velezensis* DM was retrieved from the authors’ laboratory collection, following its initial isolation from the rhizosphere of *Datura metel* L. The 16 S rRNA gene sequence of this strain has been previously submitted to the NCBI GenBank database and is available under the accession number OR364492 [6].

### Solid-state fermentation and extraction of bacterial secondary metabolites

Initial cultivation of bacterial colonies was performed on a freshly prepared LB agar plate to establish the seed culture. Subsequently, selected colonies were aseptically inoculated into a 250 mL Erlenmeyer flask containing 50 mL of liquid LB medium and incubated at 37 °C with continuous agitation at 170 rpm for 24 h [14]. Ten 1 L Erlenmeyer flasks were aseptically inoculated with 5 mL of the seed culture to initiate solid-state fermentation. Each flask contained a modified rice medium, composed of 100 g of commercial rice and 150 mL of distilled water, supplemented with 0.4% yeast extract. The bacterial cultures were then incubated under static conditions at 37 °C for 14 days. Following incubation, the cells were subjected to sonication for 30 min to facilitate bacterial cell disruption. The resulting culture medium, with a total volume of 1.5 L, was harvested and extracted through maceration with 1.5 L of MeOH. The MeOH extract was subsequently filtered under vacuum, and the aqueous MeOH filtrate was concentrated in vacuo using rotary evaporation (Heidolph, Germany) at 40 °C until all MeOH was removed. The residual material was re-suspended in water and extracted with EtOAc until exhaustion. Finally, the EtOAc extract was evaporated to dryness (yielding 24.39 g) and stored for further analysis [15].

### Chemical profiling of total bacterial extract by LC Q-TOF/MS analysis

The microbial extract was analyzed using an Agilent 1290 ultra-high-performance liquid chromatography (UHPLC) system coupled with an Agilent G6545 hybrid quadrupole time-of-flight mass spectrometer (Q-TOF/MS) (Agilent Technologies, Santa Clara, CA, USA). Chromatographic separation was performed on an Agilent Poroshell EC-C18 column (2.1 × 100 mm, 2.7 μm) maintained at 40 °C, with a flow rate of 0.2 mL/min. A gradient elution program was employed, utilizing mobile phase A (0.1% FA in water) and mobile phase B (0.1% FA in ACN). The gradient was initiated at 2% B, increased linearly to 10% B over 6 min, then further raised from 10% to 40% B over the following 9 min. Subsequently, the gradient was ramped from 70% to 100% B within 8 min, maintained at 100% B for 6 min, and then returned to 2% B over 1 min, followed by a 5-minute re-equilibration at the initial conditions. A sample volume of 2 µL of the microbial extract (1 mg/mL) was injected for analysis. The mass spectrometer was equipped with a Dual Agilent Jet Stream (AJS) electrospray ionization (ESI) source, with the following settings: nebulizer gas pressure at 50 psi, drying gas flow rate at 12 L/min with a temperature of 300 °C, and sheath gas flow rate at 11 L/min with a temperature of 350 °C. The capillary voltage (Vcap) was set at 3500 V in positive ionization mode, while the skimmer, octopole RF, and fragmentor voltages were set at 65 V, 750 V, and 130 V, respectively. Data acquisition was performed in both profile and centroid modes within the extended dynamic range (2 GHz). The instrument was calibrated and tuned according to the manufacturer’s guidelines. Accurate mass spectra were acquired over an m/z range of 100–1700, employing an MS/MS acquisition rate of 5 spectra per second. Continuous internal mass calibration was achieved using reference ions at m/z 121.0509 (protonated purine) and m/z 922.0098 (protonated hexakis (1 H,1 H,3 H-tetrafluoropropoxy) phosphazine) in positive ionization mode [16, 17].

### Data analysis of mass profile

Peak detection parameters for MS1 and MS2 data were set with mass tolerances of 0.03 Da and 0.1 Da, respectively, in centroid mode for each dataset generated from the microbial interaction profiles. Data acquisition was performed within a retention time range of 0.5 to 30 min, covering a mass-to-charge (m/z) range of 100–1700. Molecular feature extraction (MFE) was conducted with a minimum noise threshold set at 1000 counts, employing the isotope model optimized for small molecules and specialized metabolites within the batch feature extraction workflow. The charge state was restricted to 1–2, with retention time and mass windows set to ± 0.10 min and ± 0.15 ppm, respectively. A minimum MFE score of 70 was required for feature selection. For compound identification, stringent criteria were applied: a mass error below 5 ppm, peak intensity exceeding 1000 counts, confirmation by at least one co-eluting fragment ion (with a co-elution score of at least 85%), and an overall match score of 70% or higher. In positive ionization mode, the search included common adducts such as [M + H]^+^, [M + Na]^+^, and [M + NH₄]^+^, using Agilent MassHunter software. Extracted features were annotated by matching their MS/MS spectra against the METLIN metabolite database (version B.07.00), which comprises 79,609 reference spectra, and cross-referenced with an in-house spectral library constructed for the studied strain using the Agilent MassHunter PCDL Manager 8.0. In LC Q-TOF/MS chemical profiling, the error ppm value reflects the mass accuracy of detected ions, with ± 2–5 ppm generally considered acceptable for reliable identification.

### General chromatographic procedure

The EtOAc extract (24.39 g) was subjected to column chromatography using normal-phase silica gel (70–230 mesh; Merck, Germany) packed in a 60 × 3 cm glass column. Elution was performed using a gradient system of DCM and MeOH. The resulting fractions were monitored by TLC plates. Fractions exhibiting similar TLC profiles were combined and concentrated under reduced pressure using a rotary evaporator at a temperature below 40 °C.

### Purification of bacterial metabolites using chromatographic methods

A crude fraction was obtained using a solvent system of DCM: MeOH (9:1, v/v). This fraction (63.9 mg) was further purified by medium-pressure liquid chromatography (MPLC) using a Büchi system (Büchi, Switzerland) equipped with a reverse-phase C18 column (Inertsil ODS-P “5 µm, 20 × 250 mm”, GL Sciences, Tokyo, Japan). The separation was performed at a flow rate of 15 mL/min employing a gradient mobile phase of ACN/0.01% FA and water/0.01% FA. Solvent evaporation till dryness yielded the purified glycoglycerolipid **1** (6.5 mg). The purity of the isolated compound was assessed via high-performance liquid chromatography (HPLC). Qualitative HPLC analysis was conducted using an Agilent 1260-LC system equipped with an autosampler, binary pump, evaporative light-scattering detector (ELSD), and diode-array detector (DAD) (Agilent Technologies). HPLC was performed on an Inertsil ODS-3 column (5 µm, 2 × 25 cm) under isocratic conditions using a mobile phase of water: ACN (1:1, v/v) containing 0.1% FA, at a flow rate of 8 mL/min.

### Structure Elucidation of the purified compound

1D and 2D NMR spectra were recorded using a Bruker Avance DRX spectrometer (Bruker, Rheinstetten, Germany), operating at 400 MHz for ^1^H NMR and 100.40 MHz for ^13^C NMR. Deuterated MeOH (CD₃OD) was employed as the solvent for the analysis of the isolated compound. High-resolution electrospray ionization mass spectrometry (HR-ESI-MS) was performed using a quadrupole time-of-flight (QTOF) mass spectrometer (Agilent Technologies).

### Skin photoprotection MTT assay

Bacterial extract (12.5–400 µg/mL) was tested on Normal Human Dermal Fibroblast (NHDF) cells under the effect of UV in comparison with non-UV treated cells. Additionally, HaCaT cells were used to assess cytotoxicity and skin photoprotection effect of the isolated pure compound (0.6–10 µM) [18].

#### MTT assay on cells

Cells were seeded in a 96-well plate at a density of 1 × 10⁵ cells/mL using Dulbecco’s Modified Eagle Medium (DMEM) and incubated for 24 h at 37 °C in a humidified atmosphere containing 5% CO₂. After incubation, 0.5µL/ well of tested compounds at varying concentrations were added along with gallic acid as a positive control and DMSO as a negative control (at a final concentration of 0.5% v/v), using fresh serum-free medium. The cells were then incubated for an additional 24 h under the same conditions. Subsequently, the wells were washed twice with phosphate-buffered saline (PBS). Following this, 10 µL of 3-(4,5-dimethylthiazol-2-yl)-2,5-diphenyltetrazolium bromide (MTT) solution (5 mg/mL in PBS; Tokyo Chemical Industry, Tokyo, Japan) was added to each well, and the plates were incubated for a further 4 h. After incubation, the supernatant was carefully removed and replaced with 100 µL of isopropyl alcohol containing 40 mM HCl to solubilize the formazan crystals. The absorbance was then measured at 570 nm using a microplate reader (BioTek Instruments, Winooski, VT, USA).

#### UVB-induced cell damage assay

Cells were seeded into a 96-well plate at a density of 1 × 10⁵ cells/mL and incubated for 24 h at 37 °C in a humidified atmosphere containing 5% CO₂. Following incubation, 0.5µL/ well of the tested compounds at varying concentrations, along with gallic acid (used as a positive control) and DMSO (negative control at a final concentration of 0.5% v/v), were added in fresh serum-free medium and incubated for an additional 24 h. Subsequently, the cells were washed twice with phosphate-buffered saline (PBS), and 100 µL of PBS was added to each well. The cells were exposed to UVB irradiation at a dose of 300 mJ/cm² using a fluorescent lamp with a peak emission at 312 nm (Bio-Link Crosslinker, Vilber Loubert Biolink™ BLX UVB, Cedex, France). Based on the measured lamp intensity of 10 mW/cm², the corresponding irradiation time was 30 s After UVB exposure, the PBS was replaced with fresh serum-free medium, and the cells were incubated for a further 24 h. Finally, cell viability was assessed using the MTT assay as previously described.

### Anti-melanogenesis assay

The anti-melanogenic activity of the isolated compound (1.25, 2.5, and 5 µM) was evaluated using B16-F10 murine melanoma cells. Two sets of 24-well plates were seeded with cells at a density of 0.5 × 10⁵ cells/mL and incubated simultaneously for 24 h at 37 °C in a humidified atmosphere containing 5% CO₂. One set of plates was designated for melanin content analysis, while the second was used to assess cell viability via the MTT assay. Following the initial incubation, the culture medium was replaced with fresh medium containing 2µL/well from either the test compounds or DMSO as a vehicle control (with final concentration 0.2% v/v) Arbutin was included as a positive control. After 48 h of treatment, the medium was once again replaced with fresh medium containing the respective samples or DMSO, followed by an additional 24-hour incubation. For melanin content determination, cells from the first set of plates were washed with phosphate-buffered saline (PBS) and treated with 1 N NaOH to solubilize melanin at 65 °C for 1 h. Absorbance was measured at 405 nm using a microplate reader (Biotek). For the assessment of cell viability in the second set of plates, the culture supernatant was removed and cells were washed twice with PBS. Subsequently, 50 µL of MTT reagent (5 mg/mL in PBS) was added to 1.0 mL of fresh medium and dispensed into each well. Plates were incubated for 4 h at 37 °C under 5% CO₂. After incubation, the medium was aspirated and replaced with 1.0 mL of isopropyl alcohol containing 40 mM HCl to dissolve the formazan crystals. Plates were kept at room temperature in the dark for 4 h, after which absorbance was recorded at 570 nm using the same microplate reader [18].

### Anti‑allergy in vitro assay

#### Cell viability MTT assay

RBL-2H3 cells were seeded into a 96-well plate at a density of 2.5 × 10⁴ cells per well and cultured in Dulbecco’s Modified Eagle Medium (DMEM) for 24 h at 37 °C in a humidified incubator containing 5% CO₂. After incubation, the medium was replaced with fresh serum-free DMEM containing 0.5µL/ well of various concentrations of the tested compound (0.6–10 µM) or DMSO as a negative control (at a final concentration of 0.5% v/v). The cells were then incubated for an additional 24 h under the same conditions. Subsequently, 10 µL of MTT solution (5 mg/mL in phosphate-buffered saline [PBS]) was added to each well, followed by a 4-hour incubation. After the formation of formazan crystals, the supernatant was discarded and replaced with 100 µL of isopropanol containing 0.04 N hydrochloric acid (HCl) to solubilize the formazan. Cell viability was quantified based on the cellular dehydrogenase-mediated reduction of MTT to formazan, as determined by measuring absorbance at 570 nm using a microplate reader [19].

#### Determination of β‑hexosaminidase release and its inhibitory activity

RBL-2H3 cells were seeded into a 96-well plate at a density of 2.5 × 10⁴ cells/mL and incubated for 24 h at 37 °C in a humidified atmosphere containing 5% CO₂. Following incubation, the culture medium was replaced with 100 µL of Tyrode’s buffer. Subsequently, 0.5 µL of each test compound at varying concentrations (1, 5, and 10 µM), or DMSO (used as a negative control at a final concentration of 0.5% v/v), was added to the wells. Quercetin was employed as a positive control. After 1 h of incubation, 100 µL of Tyrode’s buffer containing 1% (v/v) A23187 was added to each well to induce degranulation, followed by an additional 1-hour incubation period. Thereafter, 50 µL of the supernatant was transferred to a new 96-well plate, and 50 µL of the substrate solution, *p*-nitrophenyl N-acetyl-β-D-glucosaminide prepared in citrate buffer (pH 4.5), was added to assess β-hexosaminidase release. The reaction mixture was incubated at room temperature for 1 h, after which 100 µL of sodium bicarbonate buffer (pH 10.0) was added to terminate the reaction. Absorbance was measured at 405 nm using a microplate reader to quantify the enzymatic activity [20].

### Statistical analysis

In vitro cell proliferation and viability assays were performed by evaluating the specified compound concentrations in two independent experiments, each conducted in triplicate. Data visualization and statistical analyses were carried out using GraphPad Prism software (version 8, San Diego, CA, USA). Comparisons between treated and control groups were analyzed using one-way analysis of variance (ANOVA), followed by Tukey’s post hoc multiple comparison test. Results are presented as mean ± standard deviation (SD), *n* = 3. Statistical significance was denoted as follows: *p* ≤ 0.05 (*), *p* ≤ 0.01 (**), *p* ≤ 0.001 (***), and *p* ≤ 0.0001 (****).

## Results

### LC–MS profiling

**Fig. 1:**
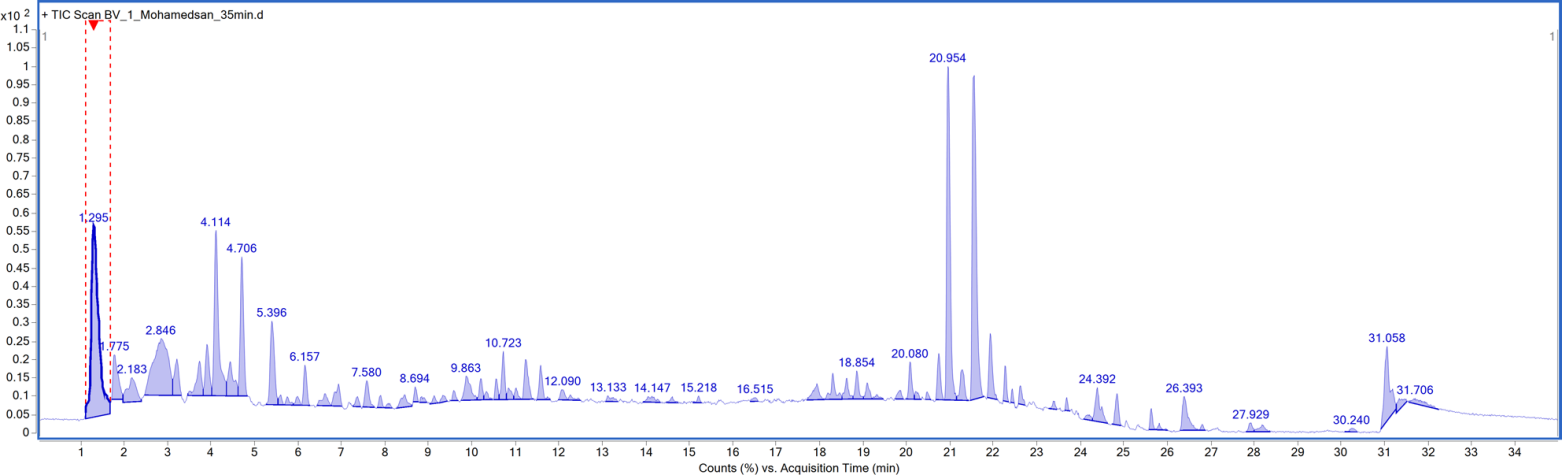
Total ion chromatogram (TIC) of bacterial crude extract derived from *Bacillus velezensis* DM

Chemical profiling of the EtOAc extract of *Bacillus velezensis* DM by using HR-LC-MS led to tentative identification of 46 metabolites divided into different chemical classes (Fig. 1 and Table S1). The compounds were distributed across several categories, comprising 16 nitrogenous compounds, ten dipeptides, ten lipid derivatives, six lipopeptides, two steroids, one miscellaneous compound, and one α-keto acid.

### Structure Elucidation of the isolated glycoglycerolipid 1

**Fig. 2:**
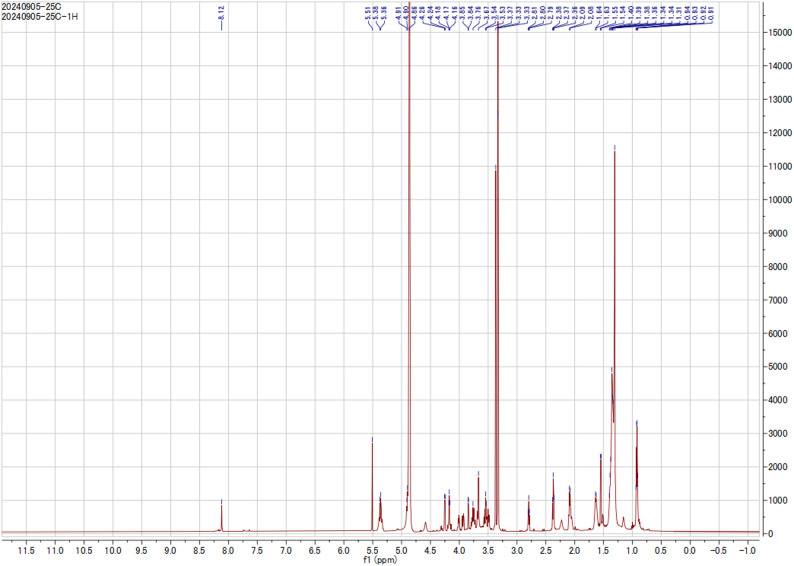
^1^H NMR (400 MHz, CD_3_OD) spectrum of a microbial glycoglycerolipid **1** isolated from *Bacillus velezensis* DM

**Fig. 3:**
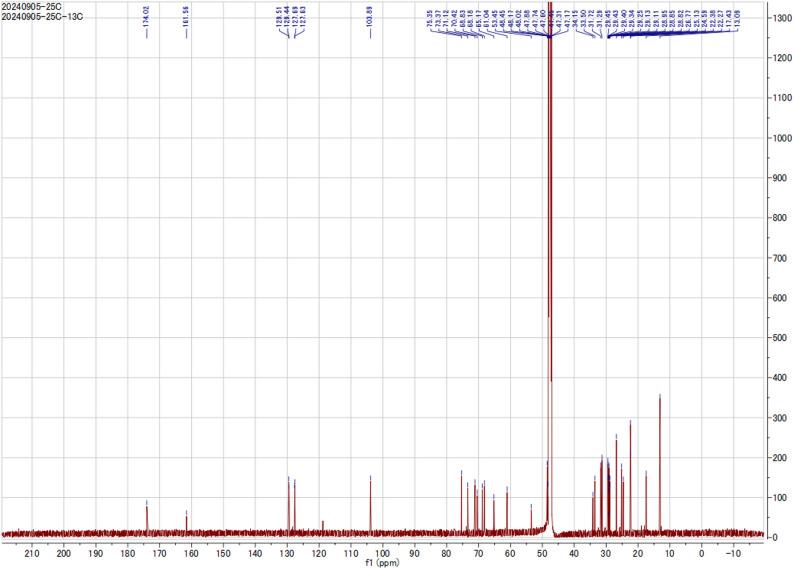
1D ^13^C NMR (100.63 MHz, MeOD), spectrum of the isolated microbial glycoglycerolipid **1**

A crude fraction, obtained from open CC of the EtOAc extract of *B. velezensis* DM, was subjected to further fractionation and purification by MPLC (as described before) to afford four subfractions (F-I to F-IV). Subfraction (F-II, eluted by 80% ACN/0.01 FA) afforded glycoglycerolipid **1** (6.5 mg) in a pure form. HPLC analysis revealed a single peak at a retention time of 31.107 min, indicating the presence of the pure glycoglycerolipid **1**. ^1^H and ^13^C-NMR chemical shift are illustrated in Figs. 2 and 3 and Table S2.

### Biological evaluation

#### Cell viability assay and protective effect of *B. velezensis* DM extract against UVB irradiation

**Fig. 4:**
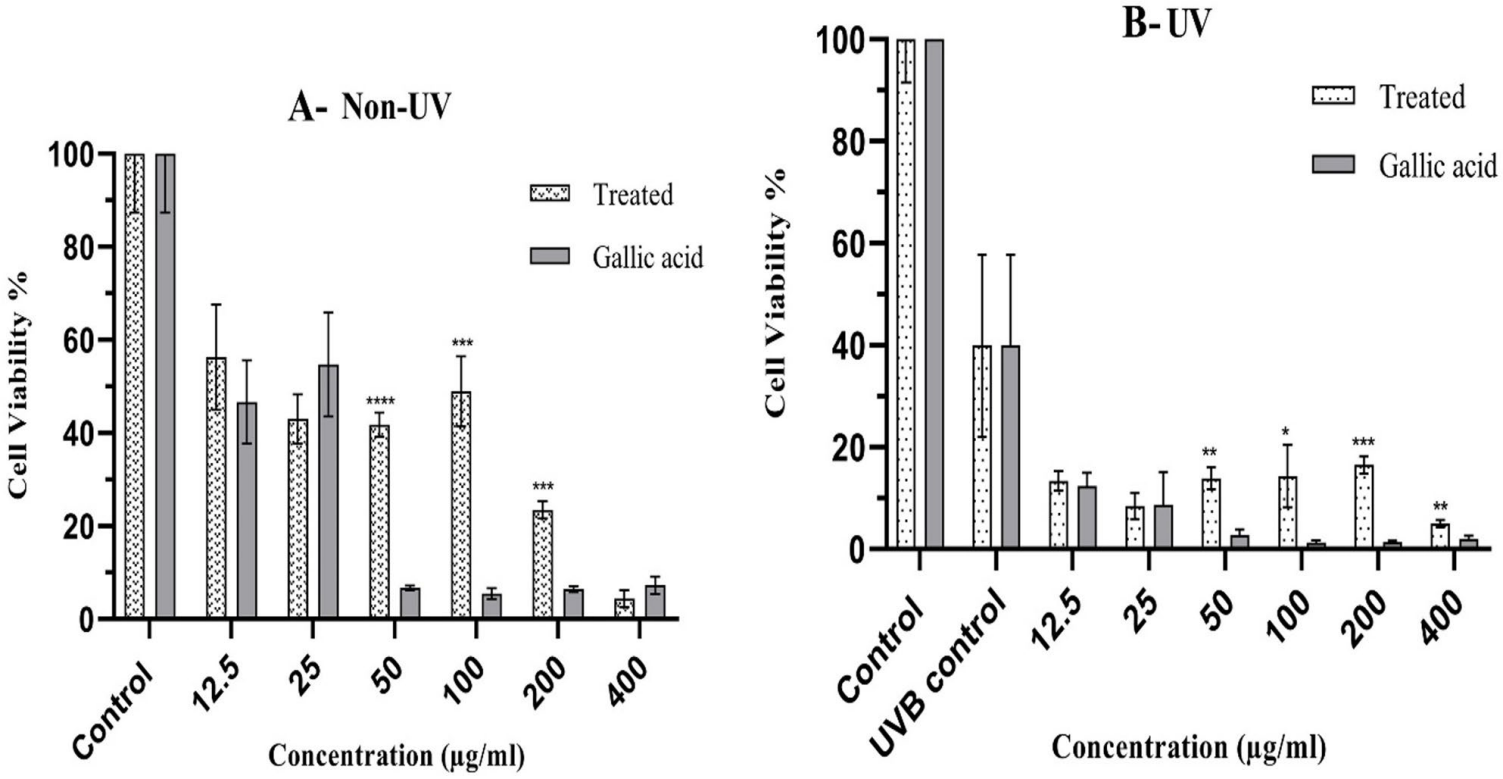
**A** Effect of *B. velezensis* DM extract (12.5–400 µg/mL) on the viability of NHDF cells. **B** Protective effect of bacterial extract against UVB irradiation in NHDF cells. UVB protection assay showed that *Bacillus velezensis* DM extract did not significantly improve cell viability in UVB-exposed NHDF cells, suggesting poor photoprotection. This may be due to a lack of ROS-scavenging activity rather than compound instability

Bacterial extract was tested on Normal Human Dermal Fibroblast (NHDF) cells under the effect of UVB in comparison with non-UVB treated cells to assess cytotoxicity. Cells were treated with bacterial extract for 5 h at different concentrations (12.5–400 µg/mL), while DMSO was used as a negative control. The results (Fig. 4) were expressed in terms of reducing yellow MTT to purple formazan by metabolically viable cells.

Regarding non-UVB treated cells, the results showed that bacterial extract exhibited high cytotoxicity at given concentrations as at the lowest concentration (12.5 µgl/mL), the cell viability was detected to be 56.3%. However, it showed the highest significant cell viability (41.8%) at a concentration of 50 µg/mL, compared to positive control (Gallic acid), which showed 6.7% cell viability at the same concentration. Followed by cell viabilities (49% and 23.5%) at concentrations 100 µg/mL and 200 µg/mL, respectively, while Gallic acid demonstrated 5.5% and 6.4% cell viabilities at the same concentrations. Lastly, the lowest cell viability found by bacterial extract was 4.4% at the highest concentration of 400 µg/mL.

On the other hand, bacterial extract did not show skin photoprotection effect at given concentrations as skin protective effect of cells treated by UVB dose at 300 mJ/cm^2^ was found to be lower than that for UVB control, showing cell viability at 39.9%. The findings revealed that at a 200 µg/mL concentration, bacterial extract demonstrated the highest cell viability (16.5%) compared to Gallic acid, which exhibited 1.4% cell viability at the same concentration. After that, at 50 µg/mL and 400 µg/mL concentrations, the strain showed cell viabilities of 13.9% and 5%, respectively, while Gallic acid displayed 2.8% and 2% cell viabilities at the same concentrations. Finally, at a 100 µg/mL concentration, it exhibited the lowest significant cell viability of 14.3% compared to Gallic acid, which showed 1.3% cell viability at the same concentration.

#### Cell viability assay and protective effect of the isolated glycoglycerolipid 1 against UVB irradiation

**Fig. 5:**
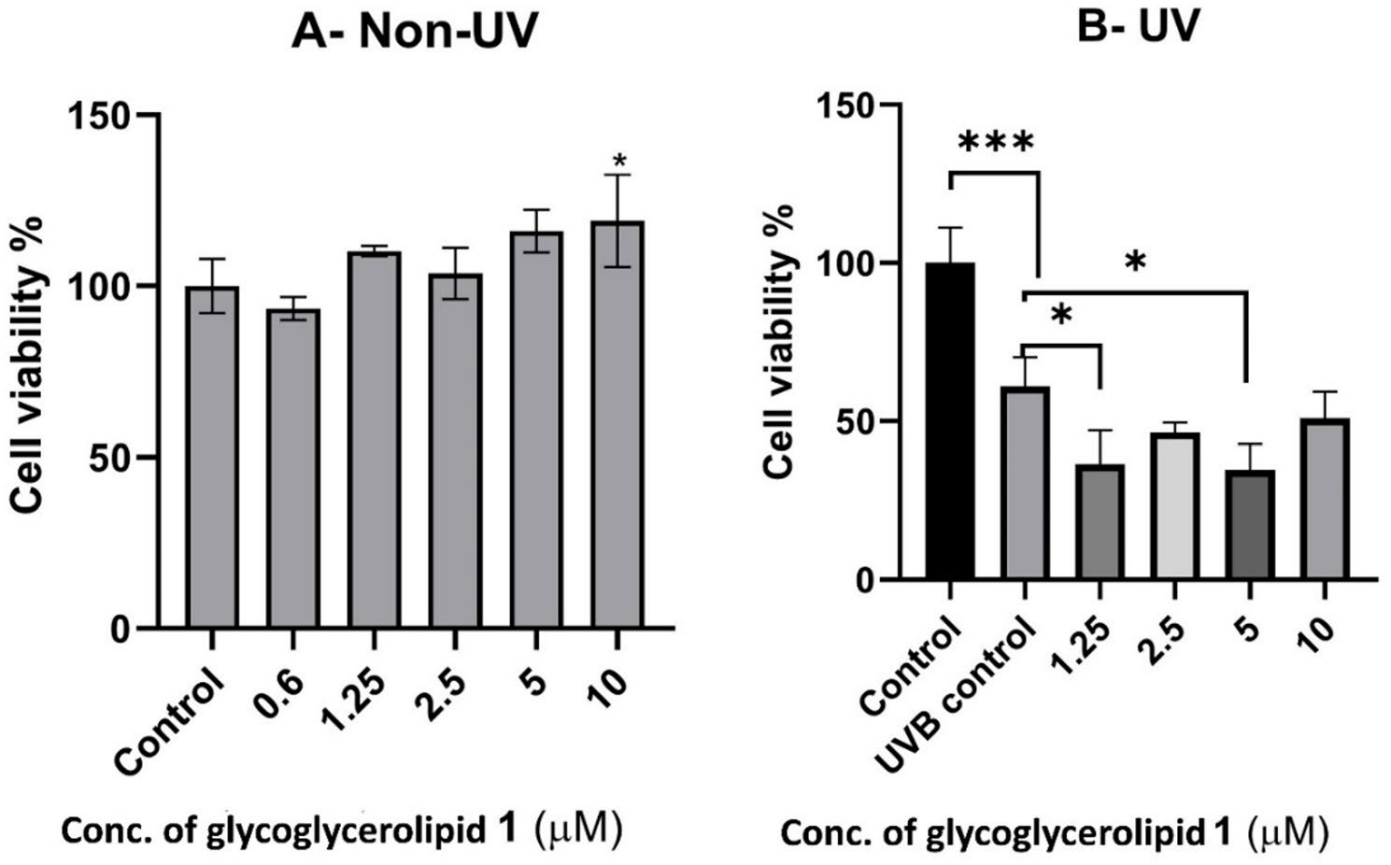
**A** Impact of the isolated glycoglycerolipid **1** (0.6–10 µM) on the viability of HaCaT keratinocytes; *p ≤ 0.05 compared with the non-treated control (DMSO). **B** Protective role of glycoglycerolipid **1** (1.25, 2.5, 5, and 10 µM) against UVB-induced damage in HaCaT cells; ***p ≤ 0.001 compared with the non-treated control, and *p ≤ 0.05 compared with UVB control group. Cytotoxicity and photoprotective assays of the isolated glycoglycerolipid **1** in HaCaT cells showed that the compound promoted cell viability but lacked photoprotective effects against UVB-induced cytotoxicity. This suggests the compound may lack ROS-scavenging activity, which is necessary for photoprotection

Figure 5a illustrates the results of a cell viability assay evaluating the cytotoxicity of glycoglycerolipid 1 isolated from *Bacillus velezensis* DM on normal HaCaT cells across various concentrations (0.6–10 µM). Notably, all glycoglycerolipid -treated groups exhibit cell viabilities at or above the control level. These results strongly suggest that the isolated glycoglycerolipid 1 is non-cytotoxic to HaCaT cells.

In addition, Fig. 5b depicts the effect of glycoglycerolipid 1 on the viability of UVB-irradiated HaCaT cells. Compared to the untreated control group (Control), which exhibited nearly 100% cell viability, the UVB control group showed a significant reduction in viability to approximately 60% (*p* < 0.001), confirming UVB-induced cytotoxicity. However, treatment with glycoglycerolipid 1 at concentrations of 1.25, 2.5, 5, and 10 µM did not result in improved cell viability compared to the UVB control as all treated groups exhibited lower viability than the UVB control. The lowest viability was observed at 5 µM, with a value below 40%, while the highest was at 10 µM, reaching around 55%. These findings suggest that glycoglycerolipid 1, at the tested concentrations, failed to exhibit any protective effect against UVB-induced damage in HaCaT cells.

#### Anti-melanogenesis assay of the isolated glycoglycerolipid 1

**Fig. 6:**
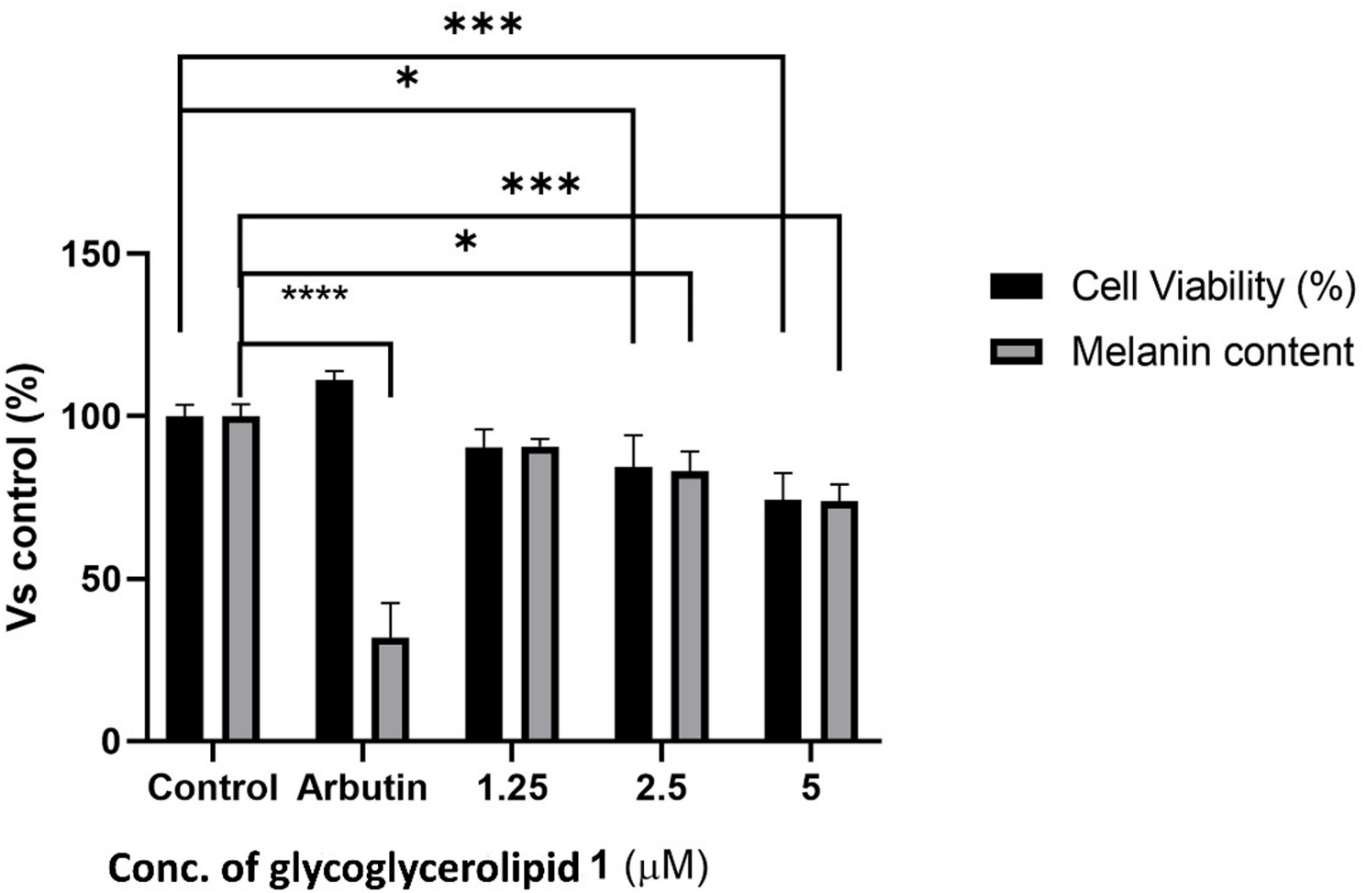
Impact of the isolated glycoglycerolipid **1** (1.25, 2.5, and 5 µM) on melanin production in B16 melanoma cells. Arbutin at a concentration of 50 µg/mL served as the positive control; *p ≤ 0.05, ***p ≤ 0.001, ****p ≤ 0.0001 compared with non-treated control (DMSO). Anti-melanogenesis assay demonstrated a dose-dependent reduction in melanin content by glycoglycerolipid **1**, but this was associated with a decrease in cell viability, suggesting the anti-melanogenic effect may be due to cytotoxicity rather than direct inhibition of melanogenesis

The anti-melanogenic activity of glycoglycerolipid **1** isolated from *Bacillus velezensis* DM was evaluated in B16 melanoma cells by assessing both cell viability and melanin content, using DMSO as the negative control and arbutin as the positive control. Initially, the compound was tested for cytotoxicity at various concentrations, and three concentrations (1.25, 2.5, and 5 µM) that maintained relatively high cell viability were selected for melanin content analysis. The compound exhibited a dose-dependent reduction in melanin content, with the greatest inhibition (27%, *p* < 0.001) observed at 5 µM (Fig. 6). However, the decrease in melanin levels closely mirrored the decline in cell viability across all tested concentrations.

#### Anti‑allergy in vitro assay

**Fig. 7:**
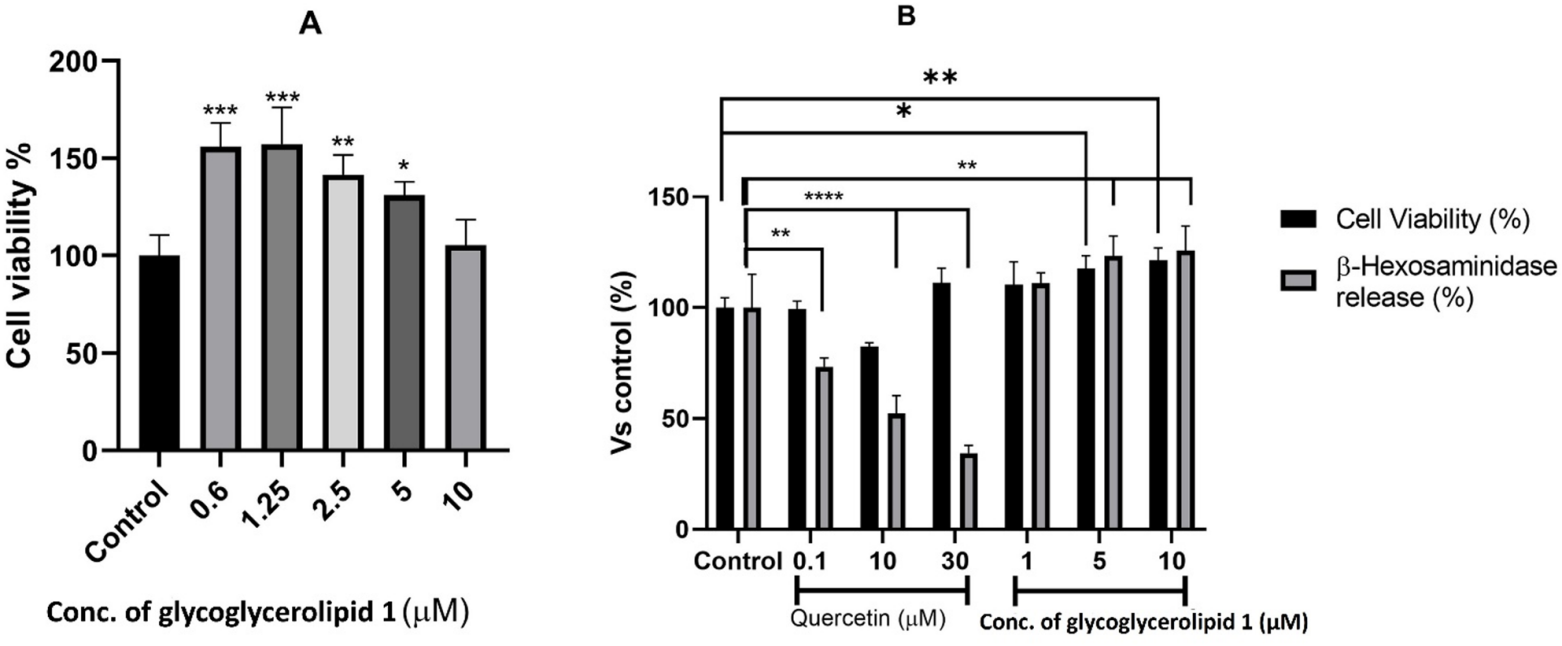
**A** The cytotoxic effect of the isolated glycoglycerolipid **1** (0.6–10 µM) on RBL-2H3 cells using a cell viability assay. **B** Anti-allergic activity in RBL-2H3 cells treated with glycoglycerolipid **1** (1, 5, and 10 µM). The anti-allergic activity was assessed by measuring the release of β-hexosaminidase following stimulation with the calcium ionophore A23187. Quercetin served as the positive control. Statistical significance was evaluated at *P < 0.05, **P < 0.01, ***p ≤ 0.001, ****p ≤ 0.0001 compared to non-treated control (DMSO). Anti-allergic assay showed that glycoglycerolipid **1** enhanced cell viability in RBL-2H3 cells but did not inhibit β-hexosaminidase release, suggesting a lack of anti-allergic activity due to its inability to modulate mast cell degranulation pathways

The anti-allergic potential of glycoglycerolipid **1** isolated from *Bacillus velezensis* DM was assessed in RBL-2H3 (rat basophilic leukemia) cells by evaluating its ability to inhibit β-hexosaminidase release and suppress mast cell degranulation following stimulation with the calcium ionophore A23187. Initially, cytotoxicity was assessed across a concentration range of 0.6–10 µM to identify concentrations that do not compromise cell viability, revealing a slight dose-dependent decrease in viability; however, even the highest concentration tested (10 µM) did not induce cytotoxicity (Fig. 7a). Based on these results, three concentrations (1, 5, and 10 µM) were selected for a subsequent experiment, which evaluated cell viability simultaneously with β-hexosaminidase release (Fig. 7b). Treatment with glycoglycerolipid **1** at these concentrations did not inhibit β-hexosaminidase release compared to the control, indicating a lack of mast cell stabilization or anti-degranulation activity.

## Discussion

### Comparative metabolomics

The chemical profiling of *Bacillus velezensis* DM using LC-MS identified a rich and diverse set of 46 bioactive metabolites, categorized into different chemical classes: nitrogenous compounds, dipeptides, lipid derivatives, lipopeptides, steroids, miscellaneous molecule and α-keto acid. Among these, nitrogenous compounds were most prevalent, including known bioactives such as tetramethylpyrazine, bacilysin, and iturin A3-A5, which are often associated with antimicrobial and plant-growth-promoting properties [9, 21]. Dipeptides like cyclo(Pro-Pro) and cyclo(Phe-Leu) are notable for their roles in microbial communication and inhibition of phytopathogens [22]. Lipid derivatives, including stearic acid and erucamide, contribute to membrane structure and signaling functions [23]. The identification of multiple lipopeptides such as surfactin A, B, and analogs further underscores the strain’s antimicrobial potential, as these compounds are well-documented for their surfactant properties and biocontrol efficacy [24, 25]. Minor classes such as steroids and unique compounds like phenyllactic acid and levulinic acid further illustrate the biosynthetic diversity of this strain, aligning with recent findings highlighting the metabolic richness and ecological roles of *Bacillus velezensis* strains in plant-microbe interactions and disease suppression [26, 27].

The chemical profiling of *Bacillus velezensis* DM presented in this study aligns closely with findings from comparative studies of other *B. velezensis* strains. In contrast, it demonstrates a broader spectrum of chemical diversity. For instance, LC-MS/MS and transcriptomics were employed to characterize *B. velezensis* CMT-6, reporting a high abundance of lipopeptides (e.g., surfactins and fengycins) under oxygen-stressed conditions, similar to the DM strain, which also produced a set of surfactins and iturins. However, the DM strain exhibited a greater diversity of nitrogenous metabolites and dipeptides, which were less emphasized in the CMT-6 strain [28]. *Bacillus velezensis* D7-8 isolated for potato disease control produced dominant lipopeptides and polyketides but lacked detailed characterization of lipid derivatives or miscellaneous compounds found in the DM profile, indicating a narrower biosynthetic scope [29]. Similarly, iturins were identified as key antifungal compounds in *B. velezensis* ZHR0, verified via LC-MS, but did not observe the steroidal and aromatic acid compounds highlighted in the DM strain [30]. Comparative molecular networking also revealed core lipopeptides across *Bacillus* spp., but highlighted metabolomic variability depending on environmental origin [31]. Collectively, these studies validate the core metabolite clusters (surfactins, fengycins, bacilysin), but the DM strain is distinct by presenting a chemically richer and more structurally varied metabolome, possibly due to unique environmental or genetic factors.

### Structure Elucidation

**Fig. 8:**
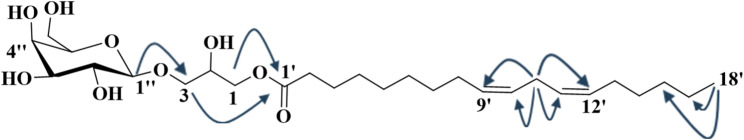
Chemical structure of a microbial glycoglycerolipid derivative isolated from *Bacillus velezensis* DM, showing observed key HMBC correlations

Glycoglycerolipid **1** was isolated as pale yellow wax. Its molecular formula was assigned to be C_27_H_48_O_9_ based on the negative HRESI-MS 551.3012 *m/z* [M + Cl]^−^ and NMR spectroscopic data (Fig. S1-S4), indicating four degrees of unsaturation. The ^13^C NMR showed three oxygenated carbons at δ_C_ 65.17, 68.18, 70.29 characteristic pattern of 1,3 disubstituted glycerol derivative. Fatty acid moiety of the isolated compound comes with full accordance with the published data and that of linoleic acid methyl ester except in the carboxyl group with three degrees of unsaturation, two for two double bonds between C-9’/C-10’, C-12’/C-13’ and one for carbonyl moiety C-1’. The previous conclusion was supported from the observed correlations between C-9/H-10 and C-13/H-12, and H_2_-11 with C-9, C-10, C-12 and C-13 in the HMBC spectrum. Additionally, the correlations between H_2_-1/C-1’ confirmed attachment of linoleic acid to C-1′ of glycerol backbone. The sugar moiety was attached to C-3 based on the observed correlations between anomeric proton of sugar H-1”/C-3 in the HMBC spectrum. The observed correlations between H-1/H-2, H-2/H-3, H-10/H-11, and H-11/H-12 in the ^1^H-^1^H COSY spectrum confirmed the suggested structure. Sugar moiety was identified as galactose based on the comparison of its NMR data with previously published paper, together with the upfield shift of C-4” in galactose [32, 33]. From the previous data, the isolated compound could be identified as:

(2 S)-1-O-(9Z,12Z-octadecadienoyl)-3-O-*β*-D-galactopyranosylglycerol (Fig. 8). According to the previous data, showing structure elucidation of the isolated compound. This study represents the first report of the isolation and structural characterization of this compound from *Bacillus velezensis* DM.

### Mechanistic insights

The biosynthesis of the isolated glycoglycerolipid [(2 S)-1-O-(9Z,12Z-octadecadienoyl)-3-O-β-D-galactopyranosylglycerol] is likely associated with the fatty acid and glycosylation machinery typical of *Bacillus velezensis*. Genomic studies have revealed that this species harbors multifunctional gene clusters encoding nonribosomal peptide synthetases (NRPS), polyketide synthases (PKS), and glycosyltransferases, which collectively support the biosynthesis of diverse metabolites such as surfactins, iturins, and fengycins [9, 24, 30]. The glycoglycerolipid structure suggests a biosynthetic route involving the esterification of a polyunsaturated fatty acid (e.g., linoleic acid) to a glycerol backbone, followed by glycosylation catalyzed by UDP-dependent glycosyltransferases [34]. This pathway likely shares intermediates and enzymatic homology with surfactin and iturin biosynthesis, where fatty acyl-CoA precursors are incorporated through NRPS/PKS systems. Such overlap indicates that the metabolic diversity of *B. velezensis* DM may result from regulatory cross-talk between lipid and peptide biosynthetic clusters, potentially influenced by rhizospheric environmental factors [17].

The comparative study of cell viability and UVB protective effects of *Bacillus velezensis* DM extract reveals its cytotoxic effect with limited photoprotection. Gallic acid was used primarily as a comparative antioxidant control due to its well-documented ROS-scavenging activity. However, it is recognized that gallic acid might exert photosensitizing and cytotoxic effects under UV exposure. Future studies will incorporate recognized photoprotective compounds such as ascorbic acid, niacinamide, or ferulic acid for more accurate benchmarking. The bacterial extract exhibited significant cytotoxicity at all tested concentrations under non-UVB conditions, which likely masked any potential photoprotective effects. The photoprotective potential of the extract at sub-cytotoxic concentrations remains undetermined. Future investigations will evaluate a broader concentration range, beginning from the lowest non-toxic doses (≤ 6.25 µg/mL) to ensure biologically valid interpretation. These outcomes might come from inherent metabolic stress or oxidative effects of bioactive compounds in the extract. Unlike other *B. velezensis* strains, such as MTCC13097, which produces UV-protective sulfated polysaccharides like mannogalactans that significantly reduce matrix metalloproteinase (MMP) expression and DNA fragmentation under UV exposure [35], the DM extract might lack such specialized components or present them in sub-therapeutic quantities. Moreover, similar work on *B. velezensis* G7 demonstrated effective UV shielding via pigment-like compounds and bacteriocin expression, reinforcing that the efficacy is highly strain-dependent [36]. Photoprotection in fibroblasts also depends on extract composition, cellular uptake, and antioxidant balance. Compounds like fengycin and surfactin found in *B. velezensis* may contribute to UV resistance but can also disrupt membranes and induce cytotoxicity at high doses [37]. Therefore, the extract’s mixed effects could reflect a complex interplay of protective and damaging agents.

The isolated glycoglycerolipid compound from *Bacillus velezensis* DM showed non-cytotoxic behavior in HaCaT keratinocytes, enhancing cell viability across all tested concentrations. This result suggests proliferative or metabolic-stimulating activity under non-stress conditions. However, the same compound failed to protect HaCaT cells from UVB-induced cytotoxicity, as cell viability remained below the UVB control group. This contrast implies that while the glycoglycerolipid compound may enhance basal cellular activity, it lacks the antioxidant, DNA-stabilizing, or anti-inflammatory properties necessary for UVB photoprotection. Similar findings investigated surfactin, a lipopeptide from *Bacillus velezensis*, noting its stimulation of keratinocyte proliferation in normal conditions but pro-apoptotic effects under UVB exposure due to membrane permeabilization and oxidative imbalance [38]. Additionally, glycolipids and surfactants often interact with cellular membranes, and in oxidative environments like UVB irradiation, they can aggravate lipid peroxidation or mitochondrial disruption if not balanced by antioxidants [39]. Unlike sulfated polysaccharides from other *B. velezensis* strains, which show strong UV-blocking and DNA-protective effects [35], the glycoglycerolipid tested here may lack structural features essential for UVB shielding or free radical scavenging. Other studies, such as those on glycosylated natural products, suggest that UV-protective efficacy in keratinocytes relies on modulating pathways like MAPK, NRF2, or inhibiting MMPs [40]. The lack of photoprotection may also be due to suboptimal intracellular uptake, inadequate compound stability under UVB, or pro-oxidative transformation products [41]. The absence of effective photoprotection could be due to the inability of the isolated compound to interact with key cellular pathways involved in UVB protection, such as antioxidant defense mechanisms (e.g., Nrf2 signaling) or DNA repair processes.

Although the current photoprotection assay evaluated cell viability as a primary endpoint, additional mechanistic validation through ROS and MMP-level analyses would strengthen the interpretation. Measuring intracellular ROS accumulation could help determine whether the lack of UVB protection is due to insufficient antioxidant or ROS-scavenging activity, as reported for other *Bacillus*-derived metabolites such as surfactins and fengycins [18, 24]. Likewise, quantifying MMP-1 and MMP-9 expression would clarify whether the compound modulates extracellular matrix degradation and photoaging processes commonly triggered by UVB stress [5]. Incorporating these assays in future studies would better elucidate the biochemical basis of the compound’s photobiological behavior.

The glycoglycerolipid compound derived from *B. velezensis* DM showed considerable anti-melanogenesis potential by reducing melanin content. The melanin suppression trend closely mirrored a decrease in cell viability. This parallel trend suggests that the observed inhibition of melanogenesis may be primarily due to cytotoxic effects, rather than a direct suppression of melanin production in viable cells. Therefore, the anti-melanogenic activity of the glycoglycerolipid compound cannot be conclusively separated from its cytotoxic impact under the tested conditions. This bioactivity aligns with prior findings on microbial glycolipids’ capacity to affect pigmentation pathways, especially in melanogenesis models. For example, studies on microbial glycolipids such as rhamnolipids and sophorolipids have reported their bioactive potential in skincare applications due to antioxidant and signaling-modulatory effects that impact melanin synthesis pathways [42, 43]. The relatively moderate inhibition seen in the present results compared to potent agents like Arbutin may be due to the glycolipid’s specific mode of action, potentially modulating melanogenesis via mild suppression of tyrosinase activity or upstream signaling cascades. In addition, microbial-derived glycolipids have shown interaction with melanin-related signaling, including effects on MAPK and MITF pathways, as observed in other natural product studies [44]. The lack of higher inhibition levels at 5 µM may also be due to insufficient intracellular accumulation or lower binding affinity to melanogenesis targets. Lastly, the compound’s favorable biocompatibility marks it as a promising agent for safe, mild skin-whitening applications, potentially enhanced through formulation strategies or synergistic combinations.

The marked increase in RBL-2H3 cell viability suggests that the glycoglycerolipid compound may enhance mitochondrial function or reduce apoptotic signaling, contributing to cellular resilience. This suggests a proliferative or protective effect on mast cells, consistent with previous studies showing that glycolipids, particularly rhamnolipids and sophorolipids, can exhibit low cytotoxicity and sometimes even promote cellular viability depending on structure and purity [45, 46]. However, the absence of any effect on β-hexosaminidase release indicates that the compound does not modulate the allergic response pathway under the tested conditions. In contrast, quercetin, a well-established flavonoid with mast cell-stabilizing properties, significantly reduced enzyme release in a dose-dependent manner (**p < 0.01), aligning with its known efficacy in suppressing allergic responses [47]. The limited anti-allergic effect of the glycolipid may be attributed to several factors, including inadequate intracellular uptake, inability to interfere with calcium-mediated signaling pathways involved in degranulation, or structural features that do not favor binding to key enzymes or receptors [48]. Structural modification of the glycolipid may be necessary to enhance its biological activity. In addition, the anti-allergy potential of microbial glycolipids remains underexplored, with few reports documenting their efficacy against mast cell-mediated reactions, highlighting a need for deeper mechanistic studies [49]. To date, there are no published studies specifically addressing the dermatoprotective potential of the isolated glycoglycerolipid **1**. A previous study isolated and structurally identified several glycoglycerolipids from *Cibotium barometz*, including (2 S)-1-O-(9Z,12Z-octadecadienoyl)-3-O-β-D-galactopyranosylglycerol, and highlighted its potential pharmacological activity in bone health through the inhibition of osteoclast formation in mouse bone marrow macrophages, suggesting its role in modulating bone metabolism [50].

The lack of anti-allergic activity could be linked to the compound’s inability to modulate mast cell degranulation pathways or the inhibition of key enzymes like β-hexosaminidase, which are involved in allergic responses. In addition, the structure of the glycoglycerolipid compound might limit its interaction with these critical biological pathways. It may also be useful to examine the stability of the compound under UVB exposure or whether the compound’s concentration was suboptimal for inducing the desired effects. Furthermore, a comparison with other similar compounds in the literature could shed light on possible modifications to improve the compound’s effectiveness.

It is essential to consider how the structural features of key metabolites directly influence their biological functions. The strain produced a diverse array of metabolites, including nitrogenous compounds, dipeptides, lipopeptides, and lipid derivatives, each of which may contribute to distinct bioactive profiles. For example, dipeptides like cyclo(Pro-Pro) and cyclo(Phe-Leu) could play roles in microbial signaling or antimicrobial activity, which align with the strain’s potential in plant growth promotion and biocontrol. Lipid derivatives such as stearic acid and erucamide may be involved in membrane integrity, affecting cell interactions and biofilm formation, which could explain the strain’s potential in skin care applications. The presence of lipopeptides like surfactin suggests significant antimicrobial and surfactant properties, potentially enhancing the strain’s ability to modulate inflammation or interact with skin cells. However, linking this chemical diversity to the observed anti-melanogenesis, anti-allergic, and cytotoxic effects requires further structural optimization and mechanistic studies to pinpoint which metabolites or combinations are responsible for these activities. By using techniques like structure-activity relationship (SAR) analysis, we can better understand how the chemical structure of these metabolites determines their functional specificity, offering valuable insights for developing targeted, safe, and effective dermatological or cosmeceutical applications.

The isolated glycoglycerolipid [(2 S)-1-O-(9Z,12Z-octadecadienoyl)-3-O-β-D-galactopyranosylglycerol] differs functionally from classical microbial glycolipids such as rhamnolipids, sophorolipids, and surfactins, which typically show strong surfactant, antimicrobial, and ROS-scavenging activities due to their high amphiphilicity and multimeric sugar or peptide structures [51–53]. In contrast, the glycoglycerolipid from *Bacillus velezensis* DM demonstrated cytoprotective effects without significant photoprotective or anti-allergic activity, possibly reflecting its moderate amphiphilicity and limited redox potential. Structurally, the single galactose moiety and unsaturated linoleoyl chain may restrict hydrogen bonding and membrane interaction compared to di- or tri-saccharide glycolipids [32]. To enhance its dermatoprotective potential, structural optimization could involve introducing polar substituents (e.g., hydroxyl or sulfate groups) on the sugar headgroup or modifying the fatty acid tail to adjust hydrophilic–lipophilic balance and antioxidant reactivity. Similar modifications have been reported to enhance bioactivity and cellular compatibility in surfactin and rhamnolipid derivatives [54, 55]. Such structure activity guided optimization may improve the compound’s efficacy in cosmeceutical applications.

## Conclusion

A microbial glycoglycerolipid, [(2 S)-1-O-(9Z,12Z-octadecadienoyl)-3-O-β-D-galactopyranosylglycerol], was successfully isolated and structurally characterized for the first time from *Bacillus velezensis* DM. This finding expands the known chemical diversity of the species, revealing its capacity to produce glycolipid-type metabolites in addition to typical lipopeptides such as surfactins and iturins. While photoprotective activity of bacterial extract could not be determined under the present experimental conditions due to its high cytotoxicity to NHDF cells at tested doses, the purified glycoglycerolipid was non-toxic to keratinocytes and enhanced their viability. However, it did not protect against UVB-induced damage. The compound exhibited a dose-dependent reduction in melanin production, but this effect paralleled a decline in cell viability, indicating that the anti-melanogenic activity may be primarily due to cytotoxicity rather than direct melanogenesis inhibition. Additionally, despite enhancing mast cell viability, the compound did not suppress β-hexosaminidase release and therefore lacked anti-allergic efficacy. The findings provide a foundation for future investigations into the biosynthetic mechanisms and structure–function relationships of glycolipids within the *Bacillus* genus, offering new insights into their ecological and biochemical roles.

## Supplementary Information

Below is the link to the electronic supplementary material.

Supplementary Material 1

## Data Availability

The dataset generated or analyzed during the current study is available in the NCBI GenBank database with the accession number: OR364492.

## Abbreviations

ACN: Acetonitrile
CC: Column Chromatography
DAD: Diode Array Detector
DCM: Dichloromethane
DMEM: Dulbecco’s Modified Eagle Medium
ELSD: Evaporative Light Scattering Detector
EtOAc: Ethyl Acetate
DMSO: Dimethyl sulfoxide
FA: Formic Acid
HaCaT: Human adult low calcium temperature keratinocytes
HR-ESI-MS: High-Resolution Electrospray Ionization Mass Spectrometry
HR-LC-MS: High-Resolution Liquid Chromatography–Mass Spectrometry
LB: Luria–Bertani (medium)
MeOH: Methanol
MPLC: Medium-Pressure Liquid Chromatography
MTT: 3-(4,5-Dimethylthiazol-2-yl)-2,5-diphenyltetrazolium bromide
NHDF: Normal Human Dermal Fibroblasts
PBS: Phosphate-Buffered Saline
PCDL: Personal Compound Database and Library
PGPR: Plant Growth-Promoting Rhizobacteria
Q-TOF/MS: Quadrupole-Time of Flight Mass Spectrometry
RBL-2H3: Rat Basophilic Leukemia Cell Line
TIC: Total Ion Chromatogram
UVB: Ultraviolet B Radiation
UHPLC: Ultra-High-Performance Liquid Chromatography
