# Supplementary material for: Metabolite profiling of Bacillus velezensis DM extract linked to the rhizosphere of Datura metel L. and dermatoprotective potential of an isolated glycoglycerolipid
Source: Microb Cell Fact. 2026 Feb 2;25:42. doi: 10.1186/s12934-026-02927-w (PMC12903210; doi:10.1186/s12934-026-02927-w)
Supplement: Supplementary file 1 — Supplementary Material 1 [file 12934_2026_2927_MOESM1_ESM.docx]

**Metabolite Profiling of *Bacillus velezensis* DM Extract Linked to the Rhizosphere of *Datura metel* L. and Dermatoprotective Potential of an Isolated Glycoglycerolipid**

Mohamed A. Awad^1^

[mohamed.awad@science.sohag.edu.eg](mailto:mohamed.awad@science.sohag.edu.eg)

Sherif F.Hammad^2,8^

sherif.hammad@ejust.edu.eg

Fahd M. Abdelkarem^3^

dr.fahd@azhar.edu.eg

Amira Elkattan^4,5^

amiraabdalla@mans.edu.eg

Samir F. El-Mashtoly^6^

Samir.Elmashtoly@leibniz-ipht.de

Hesham S. M. Soliman^7,8*^

[hesham.soliman@ejust.edu.eg](mailto:hesham.soliman@ejust.edu.eg)

Kuniyoshi Shimizu^4,9^

shimizu.kuniyoshi.381@m.kyushu-u.ac.jp

^1^Botany and Microbiology Department, Faculty of Science, Sohag University, Sohag 82524, Egypt.

^2^Department of Pharmaceutical Chemistry, Faculty of Pharmacy, Helwan University, Ain‑Helwan, Cairo 11795, Egypt.

^3^Department of Pharmacognosy, Faculty of Pharmacy, Al-Azhar University, Assiut 71524, Egypt.

^4^Department of Agro-Environmental Sciences, Graduate School of Bioresource and Bioenvironmental Sciences, Kyushu University, Fukuoka 819-0395, Japan.

^5^Department of Pharmacognosy, Faculty of Pharmacy, Mansoura University, Mansoura 35516, Egypt.

^6^Leibniz Institute of Photonic Technology, Albert-Einstein-Straße, 07745 Jena, Germany.

^7^Department of Pharmacognosy, Faculty of Pharmacy, Helwan University, Ain‑Helwan, Cairo 11795, Egypt.

^8^Faculty of Pharmacy, Egypt-Japan University of Science and Technology (E-JUST), New Borg El-Arab City, Alexandria 21934, Egypt.

^9^Kyushu University Institute for Asian and Oceanian Studies, Fukuoka 819-0395, Japan

* Corresponding author: [hesham.soliman@ejust.edu.eg](mailto:hesham.soliman@ejust.edu.eg)

**Table S1** Chemical profiling of *Bacillus velezensis* DM metabolites tentatively identifies different chemical classes using HR-LC-MS.

| **No** | **Compound** | **Molecular formula** | **Rt. (min)** | **m/z** | **Ion species** | **Error ppm** |
| --- | --- | --- | --- | --- | --- | --- |
| **Nitrogenous compounds** | | | | | | |
| 1 | Tetramethylpyrazine | C_8_H_12_N_2_ | 8.356 | 137.1072 | [M+H]^+^ | -0.66 |
| 2 | 11E,12-dihydro-2Samino-11,15- hexadecadien-3R-ol | C_16_H_33_NO | 23.955 | 256.2636 | [M+H]^+^ | 0.61 |
| 3 | L-2-Aminobutyric acid | C_4_H_9_NO_2_ | 1.31 | 104.0704 | [M+H]^+^ | 0.25 |
| 4 | Hypoxanthine | C_5_H_4_N_4_O | 2.958 | 137.0454 | [M+H]^+^ | -1.28 |
| 5 | Trimethobenzamide | C_21_H_28_N_2_O_5_ | 3.015 | 411.1878 | [M+Na]^+^ | -2.14 |
| 6 | 2-Piperidinone | C_5_H_9_NO | 4.466 | 100.0756 | [M+H]^+^ | 0.17 |
| 7 | Dihydrouracil | C_4_H_6_N_2_O_2_ | 2 | 115.0501 | [M+H]^+^ | -0.62 |
| 8 | Arecoline | C_8_H_13_NO_2_ | 9.877 | 156.1016 | [M+H]^+^ | -1.92 |
| 9 | Bacilysin | C_12_H_18_N_2_O_5_ | 7.947 | 271.1299 | [M+H]^+^ | 4.01 |
| 10 | Cadaverine | C_5_H_14_N_2_ | 5.128 | 125.1057 | [M+Na]^+^ | 7.56 |
| 11 | Iturin A3-A5 | C_49_H_76_N_12_O_14_ | 21.517 | 1074.5968 | [M+NH_4_]^+^ | -0.33 |
| 12 | 4-Nitrophenethylamine | C_8_H_10_N_2_O_2_ | 10.188 | 167.0812 | [M+H]^+^ | -2.32 |
| 13 | Tolazoline | C_10_H_12_N_2_ | 7.355 | 161.1071 | [M+H]^+^ | -2.34 |
| 14 | Cyclopentolate | C_17_H_25_NO_3_ | 4.297 | 330.1453 | [M+K]^+^ | -5.16 |
| 15 | N-salicyloylserinol | C_10_H_13_NO_4_ | 3.945 | 229.1186 | [M+NH_4_]^+^ | 1.53 |
| 16 | N,N-Dimethylaniline | C_8_H_11_N | 4.142 | 122.0962 | [M+H]^+^ | -2.72 |
| **Dipeptides** | | | | | | |
| 17 | Cyclo (Pro-Pro) dipeptide | C_10_H_14_N_2_O_2_ | 5.41 | 195.1127 | [M+H]^+^ | -0.27 |
| 18 | Cyclo(L-Leu-trans-4-hydroxy-L-Pro) | C_11_H_18_N_2_O_3_ | 8.694 | 227.1393 | [M+H]^+^ | 1.11 |
| 19 | Cyclo (Phe-Pro) dipeptide | C_14_H_16_N_2_O_2_ | 10.554 | 245.1286 | [M+H]^+^ | 0.74 |
| 20 | Cyclo (Ala-Val) dipeptide | C_8_H_14_N_2_O_2_ | 6.876 | 171.1126 | [M+H]^+^ | -1.68 |
| 21 | Cyclo (Leu-Leu) dipeptide | C_12_H_22_N_2_O_2_ | 4.734 | 227.1752 | [M+H]^+^ | -1.08 |
| 22 | Cyclo (Gly-Phe) | C_11_H_12_N_2_O_2_ | 11.259 | 205.0968 | [M+H]^+^ | -1.01 |
| 23 | Cyclo (Phe-Pro) | C_14_H_16_N_2_O | 13.415 | 229.1335 | [M+H]^+^ | 1.09 |
| 24 | Cyclo (Gly-Pro) dipeptide | C_7_H_10_N_2_O_2_ | 3.001 | 155.0814 | [M+H]^+^ | -0.81 |
| 25 | Cyclo (Phe-Leu) dipeptide | C_15_H_20_N_2_O_2_ | 12.935 | 261.1597 | [M+H]^+^ | 6.37 |
| 26 | Cyclo (Phe-Leu) dipeptide | C_15_H_20_N_2_O_2_ | 12.541 | 261.1596 | [M+H]^+^ | 2.78 |
| **Lipid derivatives** | | | | | | |
| 27 | Margaric acid | C_17_H_34_O_2_ | 18.474 | 288.2898 | [M+NH_4_]^+^ | 0.29 |
| 28 | Stearic acid | C_18_H_36_O_2_ | 19.7 | 302.3053 | [M+NH_4_]^+^ | -0.65 |
| 29 | 5-Hydroxyhexanoic acid | C_6_H_12_O_3_ | 1.281 | 150.1119 | [M+NH_4_]^+^ | -3.5 |
| 30 | trans-2-Octenoic acid | C_8_H_14_O_2_ | 2.155 | 160.1341 | [M+NH_4_]^+^ | 6.49 |
| 31 | 2-Methylglutaric acid | C_6_H_10_O_4_ | 3.621 | 164.0927 | [M+NH_4_]^+^ | 6.93 |

**Table S1** (*Continued*).

| **No** | **Compound** | **Molecular formula** | **Rt. (min)** | **m/z** | **Ion species** | **Error ppm** |
| --- | --- | --- | --- | --- | --- | --- |
| **Lipid derivatives** | | | | | | |
| 32 | 10-hydroxy-10- methyl-2-undecen-4- olide | C_12_H_20_O_3_ | 10.723 | 213.1495 | [M+H]^+^ | 2.15 |
| 33 | 5-Methoxy-1-Pentanol | C_6_H_14_O_2_ | 3.409 | 141.0895 | [M+Na]^+^ | 0.28 |
| 34 | Glycerol 1-myristate | C_17_H_34_O_4_ | 18.615 | 320.2776 | [M+NH_4_]^+^ | 5.59 |
| 35 | MG (18:2(9Z,12Z)/0:0/0:0) | C_21_H_38_O_4_ | 21.926 | 355.283 | [M+H]+ | -4.69 |
| 36 | Erucamide | C_22_H_43_NO | 26.379 | 338.3423 | [M+H]^+^ | 1.47 |
| **Lipopeptides** | | | | | | |
| 37 | Lipopeptide ND-3 | C_25_H_44_N_5_O_4_ | 20.362 | 479.348 | [M+H]^+^ | 2.68 |
| 38 | Surfactin C15 | C_53_H_93_N_7_O_13_ | 25.421 | 1036.6905 | [M+H]^+^ | -0.03 |
| 39 | Surfactin C1, 7-L-valine analog, surfactin B1, B2 | C_52_H_91_N_7_O_13_ | 23.688 | 1022.675 | [M+H]^+^ | 0.16 |
| 40 | Surfactin A | C_51_H_89_N_7_O_13_ | 24.589 | 1008.6595 | [M+H]^+^ | 0.09 |
| 41 | Surfactin B1, 4-L-alanine analog | C_50_H_87_N_7_O_13_ | 24.096 | 994.6433 | [M+H]^+^ | 0.26 |
| 42 | C12 Surfactin | C_43_H_91_N_7_O_18_ | 23.561 | 994.6433 | [M+H]^+^ | -4.99 |
| **Steroids** | | | | | | |
| 43 | 17α,21-Dihydroxy-5β-pregnane- 3,11,20-trione | C_21_H_30_O_5_ | 5.086 | 401.1746 | [M+K]^+^ | 4.37 |
| 44 | 5β-cholestan- 3α,7α,12α,24(S),27-pentol | C_27_H_48_O_5_ | 23.829 | 470.3844 | [M+NH_4_]^+^ | -1 |
| **Miscellaneous compounds** | | | | | | |
| 45 | Phenyllactic acid | C_9_H_10_O_3_ | 9.37 | 184.0974 | [M+NH_4_]^+^ | 4.18 |
| **α-keto acids** | | | | | | |
| 46 | Levulinic acid | C_5_H_8_O_3_ | 1.817 | 134.081 | [M+NH_4_]^+^ | 2.89 |

**Table S2** ^1^H and ^13^C‑NMR of the isolated microbial glycoglycerolipid **1** (δ in ppm, *J* in Hz).

| Position | δ^1^H | δ^13^C |
| --- | --- | --- |
| **Glycerol** |  |  |
| 1 | 4.18 m | 65.17 |
| 2 | 3.94 m | 68.18 |
| 3 a  b | 3.65 m  4.01 m | 70.29 |
| **Acyl** |  |  |
| 1′ | - | 174.04 |
| 2′ | 2.38, t, *J* = 7.2 Hz | 33.50 |
| 3′ | 1.63 m | 24.59 |
| 4′ | 1.31-1.36 m | 29.11 |
| 5′ | 1.31-1.36 m | 29.33 |
| 6′ | 1.31-1.36 m | 29.34 |
| 7′ | 1.31-1.36 m | 29.43 |
| 8′ | 2.04-2.1 m | 28.95 |
| 9′ | 5.32-5.40 m | 129.52 |
| 10′ | 5.32-5.40 m | 127.65 |
| 11′ | 2.80 t, *J* = 5.9 Hz | 25.13 |
| 12′ | 5.32-5.40 m | 127.65 |
| 13′ | 5.32-5.40 m | 129.52 |
| 14′ | 2.04-2.1 m | 28.8 |
| 15′ | 1.33-1.37 m | 22.38 |
| 16′ | 1.31-1.36 m | 31.29 |
| 17′ | 1.31-1.36 m | 31.71 |
| 18′ | 0.92, t *J* = 7.7 Hz | 13.09 |
| **Sugar** |  |  |
| 1′′ | 4.24, d, *J* = 7.7 Hz, | 103.90 |
| 2′′ | 3.66 | 71.12 |
| 3′′ | 3.49 | 73.37 |
| 4′′ | 3.84,d, *J* = 3.0 Hz | 68.8 |
| 5′′ | 3.54 | 75.55 |
| 6′′ | 3.75 | 61.05 |

**Structure elucidation of the isolated microbial glycoglycerolipid 1**

**
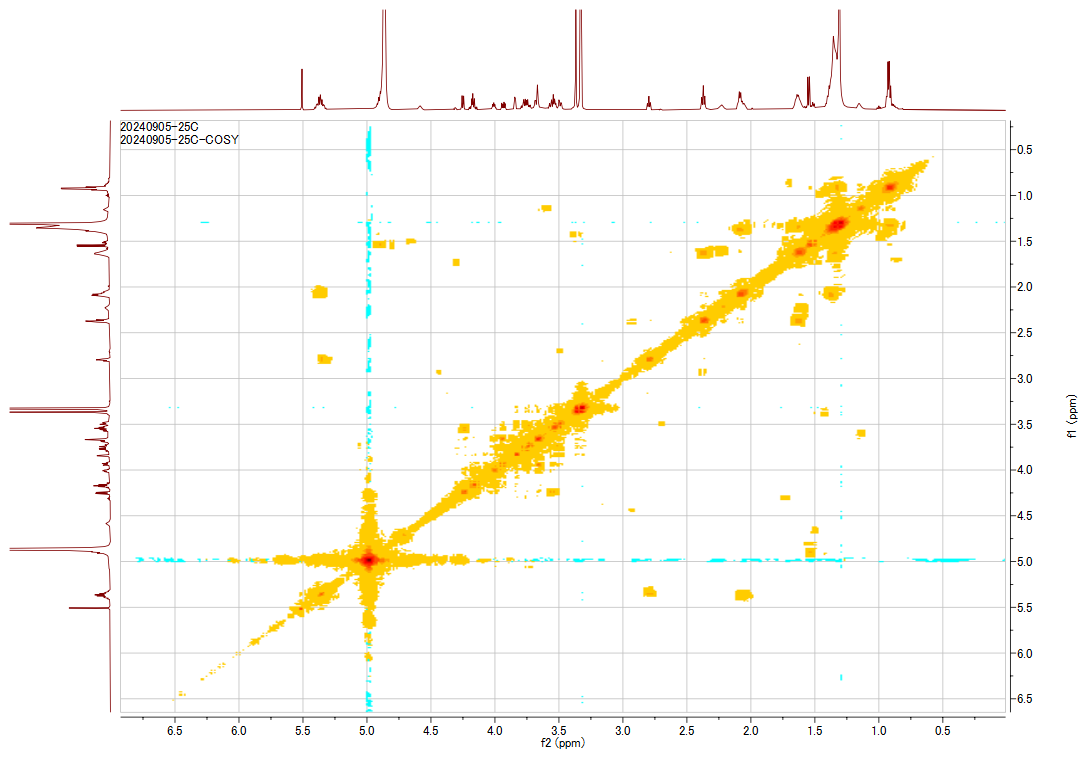
**

**Fig. S1** ^1^H-^1^H COSY spectrum of the isolated microbial glycoglycerolipid **1**.

**
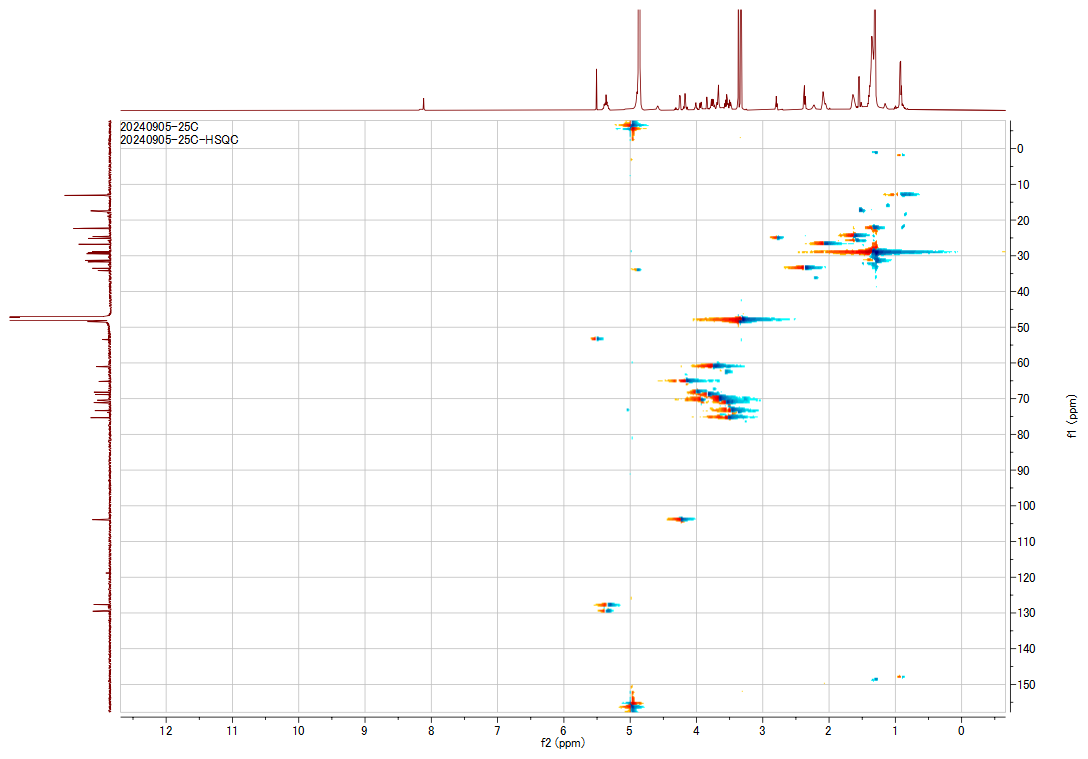
**

**Fig. S2** HSQC spectrum of the isolated microbial glycoglycerolipid **1**.

**
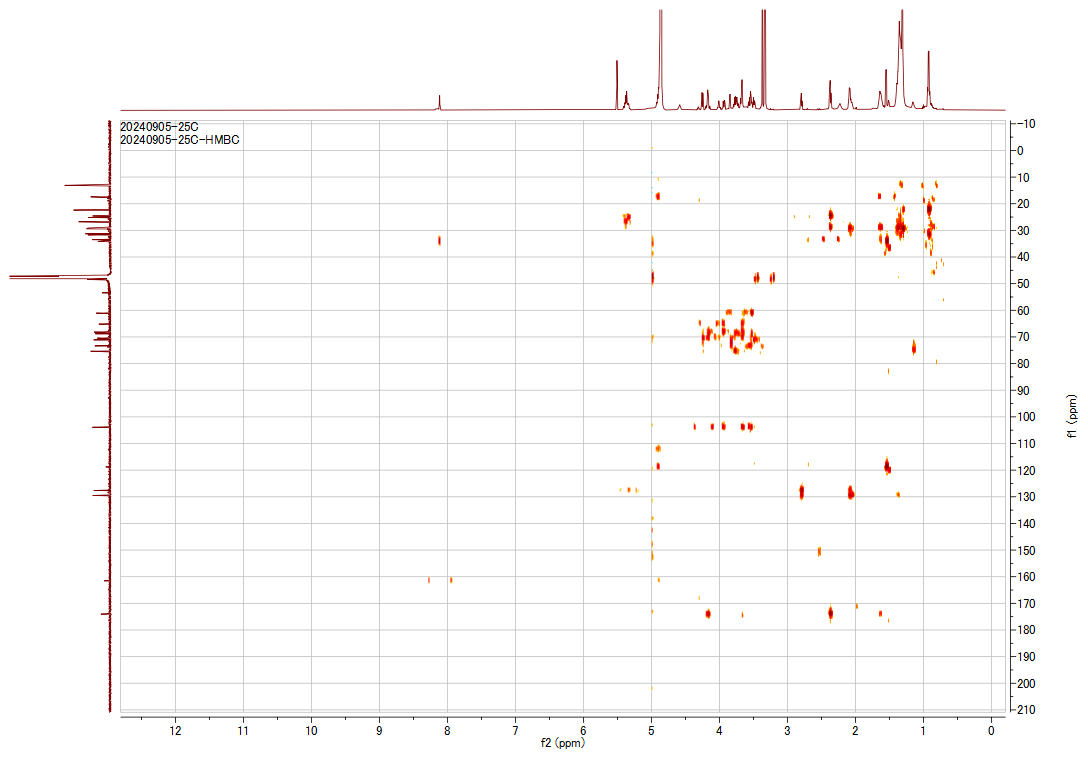
**

**Fig. S3** HMBC spectrum of the isolated microbial glycoglycerolipid **1**.

**
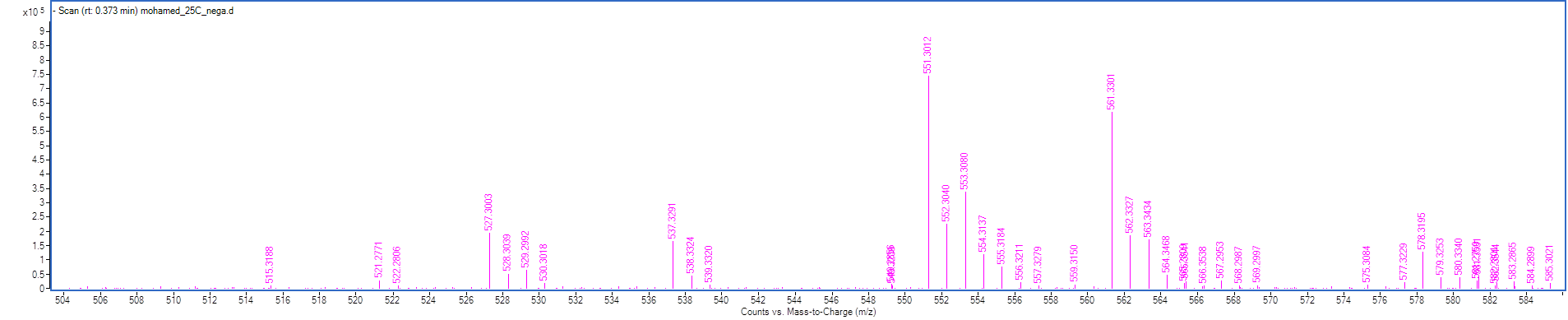
**

**Fig. S4** Negative HR-ESI-MS spectrum of the isolated microbial glycoglycerolipid **1**.

**Cell viability assay and protective effect of the bacterial extract against UVB irradiation**

| Concentration (µg/mL) | Cell viability Average (%) | SD |
| --- | --- | --- |
| Negative control | 100.0 | 12.6 |
| 12.5 | 56.3 | 11.33 |
| 25 | 43 | 5.29 |
| 50 | 41.8 | 2.55 |
| 100 | 49 | 7.53 |
| 200 | 23.5 | 1.83 |
| 400 | 4.4 | 1.8 |

**Corresponds Fig. 4A**

|  | Concentration (µg/mL) | Cell viability Average (%) | SD |
| --- | --- | --- | --- |
|  | **Negative control** | 100.0 | 16.36 |
|  | **UVB control** | 39.9 | 17.86 |
| Bacterial extract | **12.5** | 13.4 | 1.95 |
|  | **25** | 8.4 | 2.56 |
|  | **50** | 13.9 | 2.16 |
|  | **100** | 14.3 | 6.12 |
|  | **200** | 16.5 | 1.70 |
|  | **400** | 5.0 | 0.69 |

**Corresponds Fig. 4B**

**Cell viability assay and protective effect of the isolated glycoglycerolipid 1 against UVB irradiation**

| Concentration (µM) | Cell viability  Average (%) | SD |
| --- | --- | --- |
| Negative control | 100 | 7.89801 |
| gly 0.6 | 93.4426 | 3.40154 |
| gly1.25 | 110.174 | 1.50554 |
| gly 2.5 | 103.713 | 7.51613 |
| gly 5 | 116.056 | 6.26567 |
| gly 10 | 119.045 | 13.536 |

**Corresponds Fig. 5A**

|  | Concentration (µM) | Cell viability  Average (%) | SD |
| --- | --- | --- | --- |
|  | **Negative control** | 100 | 11.1718 |
|  | **UVB control** | 60.9598 | 9.25155 |
| Gly | **1.25** | 36.6236 | 10.4743 |
|  | **2.5** | 46.4261 | 3.1262 |
|  | **5** | 34.6494 | 8.15946 |
|  | **10** | 50.953 | 8.37864 |

**Corresponds Fig. 5B**

**Anti-melanogenesis assay (Corresponds Fig. 6)**

| Concentration  (µM) | Cell viability  Average (%) | SD | Melanin Content | SD |
| --- | --- | --- | --- | --- |
| Negative control | 100.0 | 3.6 | 100.00 | 3.65427 |
| Arbutin | 111.2 | 2.6 | 31.93 | 7.90000 |
| Gly 1.25 | 90.5 | 5.4 | 90.71 | 2.34061 |
| Gly 2.5 | 84.5 | 9.7 | 83.11 | 6.10917 |
| Gly 5 | 74.3 | 8.3 | 73.82 | 5.32296 |

**Anti-allergy assay**

**1-Cell viability (Corresponds Fig. 7A)**

| Concentration  (µM) | Cell viability  Average (%) | SD |
| --- | --- | --- |
| Negative control | 100 | 10.6632 |
| Gly 10 | 105.339 | 13.1413 |
| Gly 5 | 131.106 | 6.89204 |
| Gly 2.5 | 141.528 | 10.2601 |
| Gly 1.25 | 157.289 | 18.7634 |
| Gly 0.6 | 155.978 | 12.08 |

**2-Anti-allergy assay (Corresponds Fig. 7B)**

|  | Concentration  (µM) | Cell viability  Average (%) | SD | Allergy | SD |
| --- | --- | --- | --- | --- | --- |
|  | **Negative control** | 100 | 4.39109 | 100 | 15.06820431 |
| Quercetin | **0.1** | 99.3303 | 3.59897 | 73.4375 | 4.05949408 |
|  | **10** | 82.6225 | 1.64275 | 52.34375 | 8.11898816 |
|  | **30** | 111.174 | 6.621 | 34.375 | 3.580137262 |
| Gly | **1** | 110.363 | 10.346 | 110.9375 | 4.6875 |
|  | **5** | 117.695 | 5.73663 | 123.4375 | 8.87329429 |
|  | **10** | 121.431 | 5.54568 | 125.78125 | 11.07613037 |
